# Therapeutic Potential of Senolytics in Cardiovascular Disease

**DOI:** 10.1007/s10557-020-07075-w

**Published:** 2020-09-26

**Authors:** Emily Dookun, João F. Passos, Helen M. Arthur, Gavin D. Richardson

**Affiliations:** 1 Biosciences Institute, Newcastle University, Newcastle upon Tyne, NE1 3BZ UK; 2 Department of Physiology and Biomedical Engineering, Mayo Clinic, Rochester, MN USA

**Keywords:** Cardiovascular, Senescence, Senolytic, Inflammation, Remodelling, Atherosclerosis

## Abstract

Ageing is the biggest risk factor for impaired cardiovascular health, with cardiovascular disease being the leading cause of death in 40% of individuals over 65 years old. Ageing is associated with both an increased prevalence of cardiovascular disease including heart failure, coronary artery disease, and myocardial infarction. Furthermore, ageing is associated with a poorer prognosis to these diseases. Genetic models allowing the elimination of senescent cells revealed that an accumulation of senescence contributes to the pathophysiology of cardiovascular ageing and promotes the progression of cardiovascular disease through the expression of a proinflammatory and profibrotic senescence-associated secretory phenotype. These studies have resulted in an effort to identify pharmacological therapeutics that enable the specific elimination of senescent cells through apoptosis induction. These senescent cell apoptosis-inducing compounds are termed senolytics and their potential to ameliorate age-associated cardiovascular disease is the focus of this review.

## Introduction

Cellular senescence is a stable cell-cycle arrest, a cell-fate associated with multiple changes in gene expression, chromatin organisation and expression of the senescent-associated secretory phenotype (SASP) [1–3]. The SASP involves the secretion of growth factors, cytokines, chemokines and extracellular matrix factor proteases as well as an increased production of reactive oxygen species (ROS) which maintains cellular senescence in an autocrine manner and can also induce senescence in surrounding cells in a process termed the bystander effect [4–9]. Over the last 30 years, numerous studies have demonstrated that senescence accumulates in multiple cardiovascular cell lineages and is associated with cardiovascular diseases (CVD) including heart failure (HF), coronary artery disease, atherosclerosis, aortic aneurysm and vessel stenosis as reviewed elsewhere [10, 11]. Senescence was first identified to occur as a result of attrition of DNA regions, called telomeres, which are located at the end of each chromosome, a process termed replicative senescence [12–14]. Several other stressors have been shown to induce senescence including ROS, DNA damaging agents and activated oncogenes [15, 16]. In addition, we have recently shown that accumulation of persistent telomere-associated foci of DNA damage (TAF) is a major driver of cardiomyocyte senescence during ageing. Our data suggests that cardiomyocyte TAF are induced by oxidative stress, as a result of age acquired mitochondrial dysfunction, and occur independently of telomere length and cell cycle activity, providing an explanation of how senescence can accumulate in a predominately post-mitotic cardiomyocyte population [17]. Regardless of the stressors, DNA damage at telomere regions leads to activation of a persistent DNA damage response (DDR), due to the inability of telomere regions to undergo non-homologous end joining [18, 19]. This permanent DDR results in activation of either one or both the p53/p21^Cip^ or p16^Ink4a^/retinoblastoma protein cyclin-dependent inhibitor pathways in the vast majority of cells, including cardiomyocytes [17–19].

Senescence has both positive and detrimental effects in different physiological contexts and has been suggested as an example of antagonistic pleiotropy, an evolutionary hypothesis which posits that traits which are deemed beneficial to the organism’s fitness early on in life can have detrimental effects later on due to a drop in the force of natural selection [20]. For example, senescence has been shown to have beneficial effects in the context of wound healing [21], embryonic development [22, 23] and tissue repair; however, when senescent cells accumulate later in life they have been shown to contribute to tissue dysfunction in the context of ageing and age-related disorders [24]. Although senescence is clearly associated with heart disease, it was not until the development of transgenic lines enabling selective clearance of senescent cells that it was possible to demonstrate that cellular senescence promotes cardiovascular ageing. The p16-INKATTAC transgenic mouse [25, 26] contains a transgene in which the p16^Ink4a^ promoter drives expression of the FK506-binding-protein–Caspase 8. When this mouse is given the synthetic drug AP20187 it enables Caspase 8 dimerisation and apoptosis specifically in p16^Ink4a^ expressing senescent cells. Additionally, the p16-3MR mouse model which contains a truncated herpes simplex virus 1 thymidine kinase driven by the p16^Ink4a^ promotor and the p16-nitroreductase (p16-NTR) mouse in which the p16^Ink4a^ promotor drives nitroreductase allow the specific killing of p16^Ink4a^ expressing cells by ganciclovir or metronidazole administration, respectively [21, 27]. Together these mouse models have been used to demonstrate that removal of p16^Ink4a^ expressing senescent cells improves the function of aged tissue systems, including attenuating CVD and cardiac dysfunction in several disease models and during natural ageing [25, 27, 28].

**Fig. 1:**
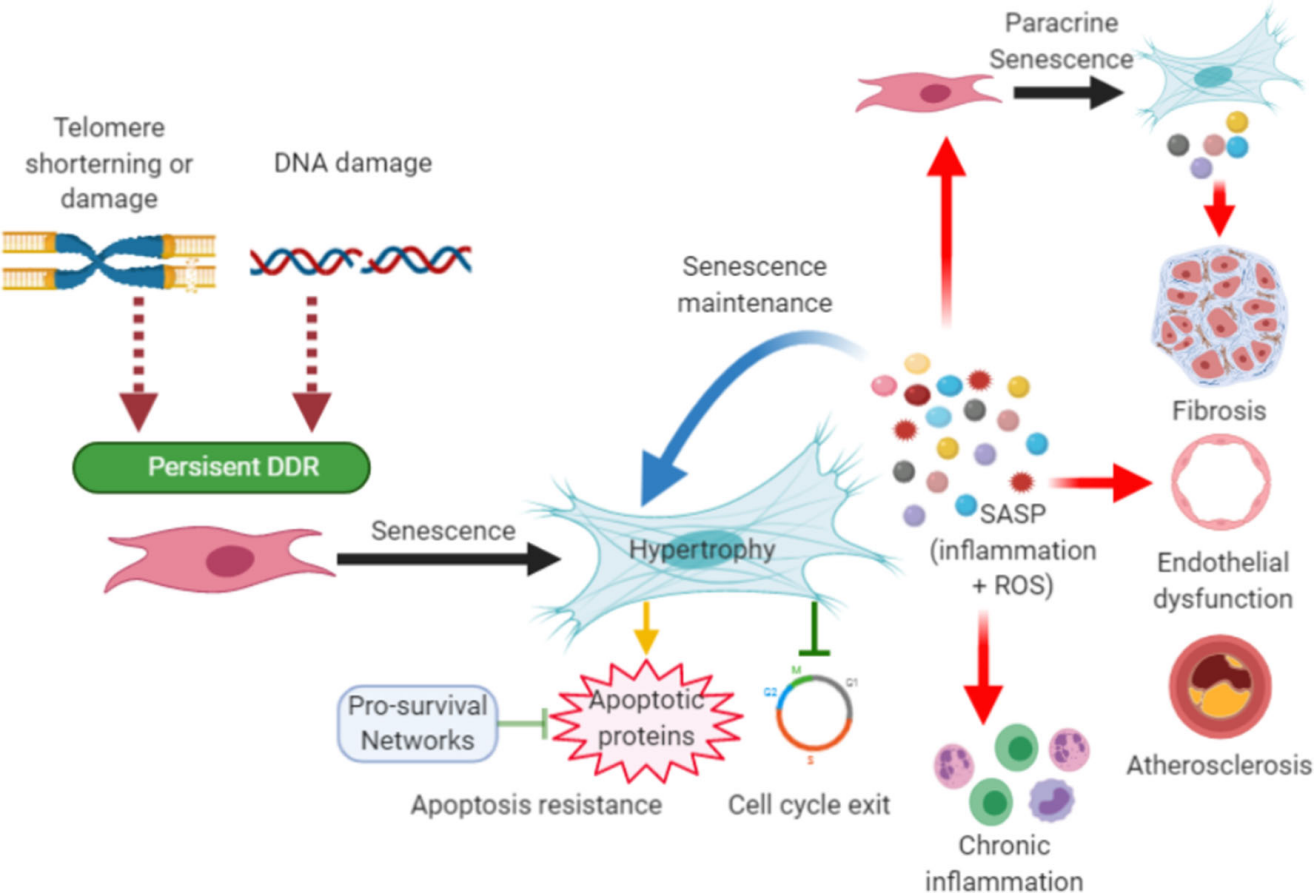
Cellular senescence and cardiovascular disease. Senescence is induced in multiple cardiovascular lineages and immune cell populations in response to a variety of stimuli including telomere attrition, telomere damage and genomic DNA damage, which leads to activation of the DDR. If the DDR is persistent, activation of tumour suppressor genes p53, p21, p16^Ink4A^ and p19^Arf^ establish cell cycle arrest and coordinate the senescence program, which can include cellular hypertrophy and expression of the SASP. Senescent cells up-regulated pro-survival pathways such as Bcl-2 and Bcl-XL that protect them from their own pro-apoptotic SASP. Cellular senescence and its associated SASP contribute to CVD including promoting inflammation, fibrosis, endothelial dysfunction and stenosis and progression of atherosclerosis. The SASP can also drive paracrine senescence in surrounding cells via the bystander effect which may further contribute to CVD pathophysiology

The striking improvement in the health of these animals has resulted in intensive investigation into pharmaceutical approaches that eliminate senescence in order to translate these findings to the clinic [17, 29–31]. Figure 1 describes an overview of the contribution of senescence to CVD as well as some of the underlying mechanisms.

## Senolytics

Senescent cells have been shown to be resistant to apoptosis via the activation of multiple pro-survival pathways [32]. Transcriptomic analysis of senescent and non-senescent pre-adipocytes, one of the most abundant senescent cells observed in vivo [33], led to the identification of several anti-apoptotic pathways that were required for senescent cell survival [31]. Activation of these pathways results in the expression or activation of several pro-survival and anti-apoptotic proteins including Bcl-2 family members, p53/p21^**Cip**^, ephrins (EFNB1 or 3), the phosphatidylinositol-4,5-bisphosphate 3-kinase delta catalytic subunit (PI3KCD), plasminogen-activated inhibitor-1 and 2 (PAI1 and 2) and hypoxia-inducible factor-1α [31, 34, 35]. Bioactive compounds that specifically target one or more of these pro-survival proteins can preferentially induce apoptosis in senescent cells and are therefore known as senolytics. Many different senolytics drugs have been identified; however, to date the Bcl-2 inhibitor navitoclax (ABT-263) [36], and a combination treatment of dasatinib (D), a tyrosine kinase inhibitor, with quercetin (Q) [31] have been best studied in the context of CVD. For that reason, we will focus on these drugs in this review.

### Dasatinib and Quercetin

Having demonstrated that siRNA mediated inhibition of components of senescent cells pro-survival networks preferentially reduced the viability of senescent cells, but not proliferating or quiescent, differentiated cells [31], Zhu et al. targeted these pathways pharmaceutically. Individually D and Q were demonstrated to have a modest senolytic activity, but in combination, D&Q treatment was an effective senolytic in vivo. D has been suggested to promote senescent cell apoptosis via the inhibition of ephrins, which regulate a pro-survival network that includes BCL-xL, PI3KCD, p21, PAI1 and PAI2 [31]. Q is a natural flavonol and an inhibitor of multiple pro-survival proteins including PAIs and PI3K [37] which when inhibited reduce Bcl-W expression [37, 38]. D&Q-mediated senescence cell clearance has been shown to attenuate lung fibrosis and hepatic stenosis, improve vasomotor function, ventricular function and neurogenesis and prevent age-related bone loss, anxiety-related behaviour and increase lifespan in pre-clinical models [31, 39–44].

### Navitoclax (ABT-263)

The observation that senescent pre-adipocytes increased expression of the Bcl-X_L_, to prevent mitochondrial-dependent apoptosis [31] led Zhu et al. to target Bcl-X_L_ with siRNA. Inhibition of Bcl-X_L_ reduced the viability of senescent but not non-senescent human umbilical vein endothelial cells (HUVEC), and subsequently, the Bcl-2 homology 3 domain (BH3) mimetic navitoclax, an inhibitor of anti-apoptotic proteins Bcl-2, Bcl-X_L_ and Bcl-W, was demonstrated to induce apoptosis in senescent human lung fibroblasts (IMR90 cell line) and HUVEC but not senescent pre-adipocytes in vitro [45]*.* Furthermore, navitoclax treatment of old mice improved the function of haematopoietic and muscle progenitor cells as assessed in ex vivo assays [46] and prevented ironising radiation-induced pulmonary fibrosis in vivo [47].

## Pharmaceutical Targeting of Senescence to Improve Myocardial Function During Ageing

The most significant determining factor of cardiovascular health is a person’s age, with CVD being the leading cause of death in 40% of individuals over 65 years [48]. The ageing heart undergoes a process of myocardial remodelling, which is characterised by physiological and molecular alterations that result in endothelial stenosis, vasomotor dysfunction and stiffening, cardiomyocyte hypertrophy, myocardial fibrosis and inflammation which result in increased ventricular stiffness, impaired cardiac function and can ultimately lead to HF [10, 11]. In particular, HF with preserved ejection fraction (HFpEF), characterised by diastolic ventricular dysfunction with maintained systolic function, is clinically associated with ageing [49]. Unfortunately, there are no proven therapies for HFpEF, and there is a growing need for novel interventions to prevent or reverse HF. The association between senescence and myocardial ageing in humans has been reported for nearly 20 years [50]. More recently it has been demonstrated that senescence contributes directly to age-related myocardial remodelling in mice, as pharmacogenetic elimination of senescent cells, using the p16-INKATTAC model, reduced myocardial fibrosis and attenuated cardiomyocyte hypertrophy [17, 25]. Elimination of senescent cells from aged p16-INKATTAC mice also increased their survival and reduced the development of cardiac dysfunction following isoproterenol-induced myocardial stress [25]. Following on from this data, we and others have hypothesised that an accumulation of senescence and the expression of a SASP drive age-related myocardial remodelling and have begun to independently investigate if senolytics can eliminate senescent cell populations resident in the aged heart in order to improve myocardial function [17, 29–31].

Zhu et al. were the first to demonstrate that pharmacological elimination of senescent cells from aged mice could improve myocardial function. Treatment of 24-month-old mice with a single dose of D&Q significantly improved left ventricular (LV) ejection fraction and fractional shortening. This observed change in function was suggested to be a result of a restoration in vascular endothelial function, although no change in smooth muscle contractile function was observed [31]. While these data suggest that senescent cells are deleterious to endothelial cell function the study did not quantify myocardial senescence prior to or following senolytics treatment. As such, it is unclear if an accumulation of senescence in the endothelial cell population or paracrine signalling via the SASP from other senescent cell populations underlies endothelial cell dysfunction.

We have shown in aged mice that senescence occurred primarily within the cardiomyocyte population and led to the expression of a cardiomyocyte-specific SASP with the potential to promote myofibroblast differentiation of fibroblasts and induce cardiomyocytes to hypertrophy in vitro [17]. In vivo, cyclical oral administration of navitoclax reduced the number of senescent cardiomyocytes, attenuated components of the cardiomyocyte SASP and reduced myocardial remodelling as indicated by a reduction in both cardiomyocyte hypertrophy and interstitial fibrosis [17, 29]. In addition, although no difference in systolic function was observed, navitoclax reduced LV mass and rescued an age-associated decline in diastolic function, both features associated with HFpEF clinically [51]. While the elimination of the SASP and a reduction in inflammation appears to contribute to navitoclax-mediated functional improvement in aged mice, cardiomyocyte regeneration may also play a role. Following the elimination of senescent cardiomyocytes, we observed a significant regenerative response shown by increased DNA replication in mononuclear cardiomyocytes and the expression of the proliferation marker aurora B kinase [17, 52]. These responses indicate true cardiomyocyte division and not simply karyokinesis, which are important to distinguish given the potential for cardiomyocytes to undergo binucleation in the absence of cell division [52–54]. Accumulation of myocardial senescence with age is not exclusive to the cardiomyocyte population. In human hearts, the SCA-1^**pos**^/c-kit^**pos**^/CD31^**neg**^CD45^**neg**^ resident cardiac progenitor cell (CPC) population also acquires a senescent phenotype with age [30]. CPC obtained from the hearts of 77–86 year-olds demonstrated a reduction in replication and reduced potential for cardiomyocyte differentiation compared with CPC obtained from younger 34–62-year-old hearts. Furthermore, following senescence induction via doxorubicin treatment, CPC expressed a SASP with a functional bystander effect. Conditioned media obtained from senescent CPC reduced the proliferative potential of healthy CPC and induced them to senescence. Unlike non-senescent CPC, senescent CPC were unable to enhance regeneration and restore cardiac function following transplantation into infarcted murine hearts [30]. Senolytic cocktail D&Q was shown to eliminate senescent CPC in vitro and similarly to the effects of navitoclax, D&Q treatment of aged mice attenuated aspects of remodelling including fibrosis and cardiomyocyte hypertrophy and promoted cardiomyocyte turnover [30]. Interestingly, D&Q also increased CPC proliferation in aged mouse hearts [30]. While there is still some debate regarding the extent by which CPC contribute to cardiomyocyte turnover during ageing, it is possible that dysfunction of this cell population may disrupt myocardial homeostasis and contribute to the observed decline in cardiomyocyte turnover that occurs with age in both humans and mice [53, 55].

Given the limited regenerative capacity of the heart, there is considerable interest in the potential of regenerative cellular therapies for the treatment of CVD such as myocardial infarction (MI) and age-related HF [56]. For cellular therapies to be effective, the grafted cells must survive, integrate, and function within the surviving myocardium. The data discussed above suggest that older age not only increases the potential for dysfunction in the very populations that are being used for cellular therapies but also increases the hostility of the recipient myocardial environment as a result of SASP mediated inflammation and the bystander effect. This may in part explain the failure of pre-clinical trials to translate clinically into regenerative therapies [30]. Preclinical studies showing successful cell regenerative therapies use young healthy animals [57, 58], whereas the prevalence of CVD increases linearly with age [59], and therefore, most patients undergoing cellular therapy are likely to display high levels of myocardial senescence [17, 60] which could create an unfavourable environment impeding incorporation and differentiation of the transplanted cell populations [30, 61]. Senolytic-mediated elimination of senescent cells from aged patients may, therefore, have the potential to improve the outcomes of such regenerative cellular therapies.

## Myocardial Infarction

The aged human population not only displays an increased prevalence of CVD but also has a poorer prognosis. For example with increasing age, there is an exponential increased in mortality as a result of MI [62] and an increased incidence of HF in those patients that survive the initial MI [63]. Similarly, aged mice demonstrate a reduced survival and cardiac function following MI compared with young animals [29, 64]. Navitoclax treatment prior to MI reduced cardiomyocyte senescence and TGF-β2 expression, a component of the cardiomyocyte SASP. Following senescence elimination, aged mice demonstrated an improved survival to MI and had a higher LV systolic function compared with control aged animals at 4 weeks post-MI [29]. In the absence of intervention aged mice demonstrated a gradual decline in LV function following MI, whereas navitoclax-treated aged mice maintained LV function, a response more comparable with the response of young mice to MI [29]. Although a detailed understanding of the mechanism requires further investigation, this data indicates that senescence contributes to impaired recovery post-MI and suggests that senolytic treatment has therapeutic potential in this disease setting. This is also in accordance with the observations that pharmacogenetic elimination of senescent cells using the p16-INKATTAC mouse model improved resistance to impaired myocardial dysfunction and improved survival following isoproterenol-induced myocardial stress [25].

While pre-existing senescence may be detrimental to recovery post-MI, senescence is also induced as a result of MI and plays a complex role in the pathophysiology of myocardial remodelling. The induction of senescence within the fibroblast population has been demonstrated to play a role in limiting cardiac fibrosis post-MI [65, 66]. Following MI, senescence accumulated in the fibroblast population and prevention of fibroblasts senescence, using a p53 knock-out mouse model, exacerbated cardiac fibrosis and increased infarct size at one-week post-MI [66]. While initially this data would suggest that elimination of senescence post-MI would only have detrimental effects, inhibition of senescence post-MI also attenuated inflammation and reduced the expression of SASP associated cytokines such as interleukin (IL) 1, chemokine ligand 1, chemokine ligand 2, IL6, granulocyte chemotactic protein 2 and macrophage colony-stimulating factor which are known inducers of fibrosis [42, 66]. Therefore, while senescence may initially limit fibrosis, if not efficiently resolved, chronic senescence could contribute to inflammation post-MI by promoting further cardiac fibrosis over an extended time frame. As senolytics eliminate senescent cells once senescence has been established, senolytic treatment provides a tool to attenuate SASP producing fibroblasts without preventing the initiation of senescence within the fibroblast population or the beneficial role fibroblast senescence plays in attenuating fibrosis following MI.

## Atherosclerosis and Vascular Senescence

Cellular senescence accumulates in the early stages of atherosclerosis, in multiple different cell types including endothelial cells, vascular smooth muscle cells, foam cells and T cells [67]. An accumulation of senescence promotes the development of atherosclerosis through multiple cell-independent mechanisms; however, common to all senescence cell types is the increase in inflammation as a result of the SASP which promotes necrotic core enlargement, extracellular matrix degeneration and cap thinning, erosion, calcification and intra-plaque angiogenesis [68]. To date, several studies have used a pharmacogenetic or a pharmacological approach to investigate the potential of targeting senescence to prevent or reverse plaque development. Using low-density lipoprotein receptor-deficient (*Ldlr*^**−/−**^) mice on a high-fat diet, a model of atherogenesis, senescence was increased in multiple cell types within the atherosclerotic lesions. Plaque-rich aortic arches had elevated transcript levels of the cyclin-dependent kinase inhibitor p16^Ink4a^ and SASP components including the matrix metalloproteases (Mmp) 3 and Mmp13 and the inflammatory cytokines *IL1α* and *TNFα* [27]*.* While this study focused primarily on the use of the p16-INKATTAC, P16-3MR and P16-NTR transgenic mouse models to eliminate senescent cells, the study also demonstrated that treatment of mice with navitoclax, after senescence was established, inhibited atherogenesis, as indicated by a reduction in plaque burden, plaque number and the average size of individual plaques [27]. Results were comparable with those observed when senescence was eliminated using a genetic mouse models, suggesting that the beneficial effects of navitoclax were a result of senescence cell clearance and not an off-target effect. Similar results were observed using an alternative model of atherogenesis with D&Q senolytic treatment. *ApoE*^***−/−***^ mice on a high-fat diet developed atherosclerotic plaques containing increased numbers of senescent cells. D&Q treatment reduced senescence burden and plaque calcification, although no difference in plaque size was observed [39].

Senescence of the T cell population has also been associated with atherogenesis and is a biomarker of CVD risk [69]. In particular, cytotoxic CD8^**+**^ T cells are pro-atherosclerotic and become functionally senescent with age, as demonstrated by both proliferative arrest and the increased production and secretion of inflammatory mediators characteristic of a SASP [70]. Once senescent, these terminally differentiated CD8^**+**^ T cells (TEMRA) are an independent predictor of all-cause mortality in the elderly [69]. In proof of principle experiments, we have investigated the effects of senolytics on immunosenescence in aged mice [69]. Navitoclax treatment rescued immunosenescence, as indicated by a decrease in CD8^**+**^ effector memory cells and an increase of the naïve CD8^**+**^ T cell population [69]. While we did not investigate the mechanisms by which senolytics increased the naïve T cells, it is possible that as well as eliminating senescent CD8^**+**^ cells, senolytic treatment influenced the dynamics of lymphocyte proliferation, as a result of reduced systemic inflammation or rejuvenation of progenitor pools, which in turn changed the balance of different T cell subpopulations.

Senescence in the regulatory T cell (T_**reg**_) population may also contribute to atherosclerosis as they can suppress the activity of proatherogenic effector T cells preventing atherosclerosis progression [71]. Activated T cells express high levels of telomerase activity to protect their telomeres from accelerated shortening, thereby evading replicative senescence [72]. We have demonstrated that increased oxidative stress suppresses T cell telomerase expression but does not affect T_**reg**_ cell proliferation [73]. We, therefore, proposed that within the atherosclerotic plaque, chronic oxidative stress and suppression of telomerase contribute to accelerate telomere attrition in the T_**reg**_ population which promotes the progression of atherosclerosis. Although the effects of senolytic therapy on the T cell populations, in the context of atherosclerosis, have yet to be investigated, clinical trials employing the small molecule activator TA-65 [74] to restore telomerase activity and prevent T cell senescence are ongoing (Telomerase Activator to Reverse Immunosenescence in Acute Coronary Syndrome: A Double-Blind, Phase II, Randomised Controlled Trial).

## Future Directions

**Fig. 2:**
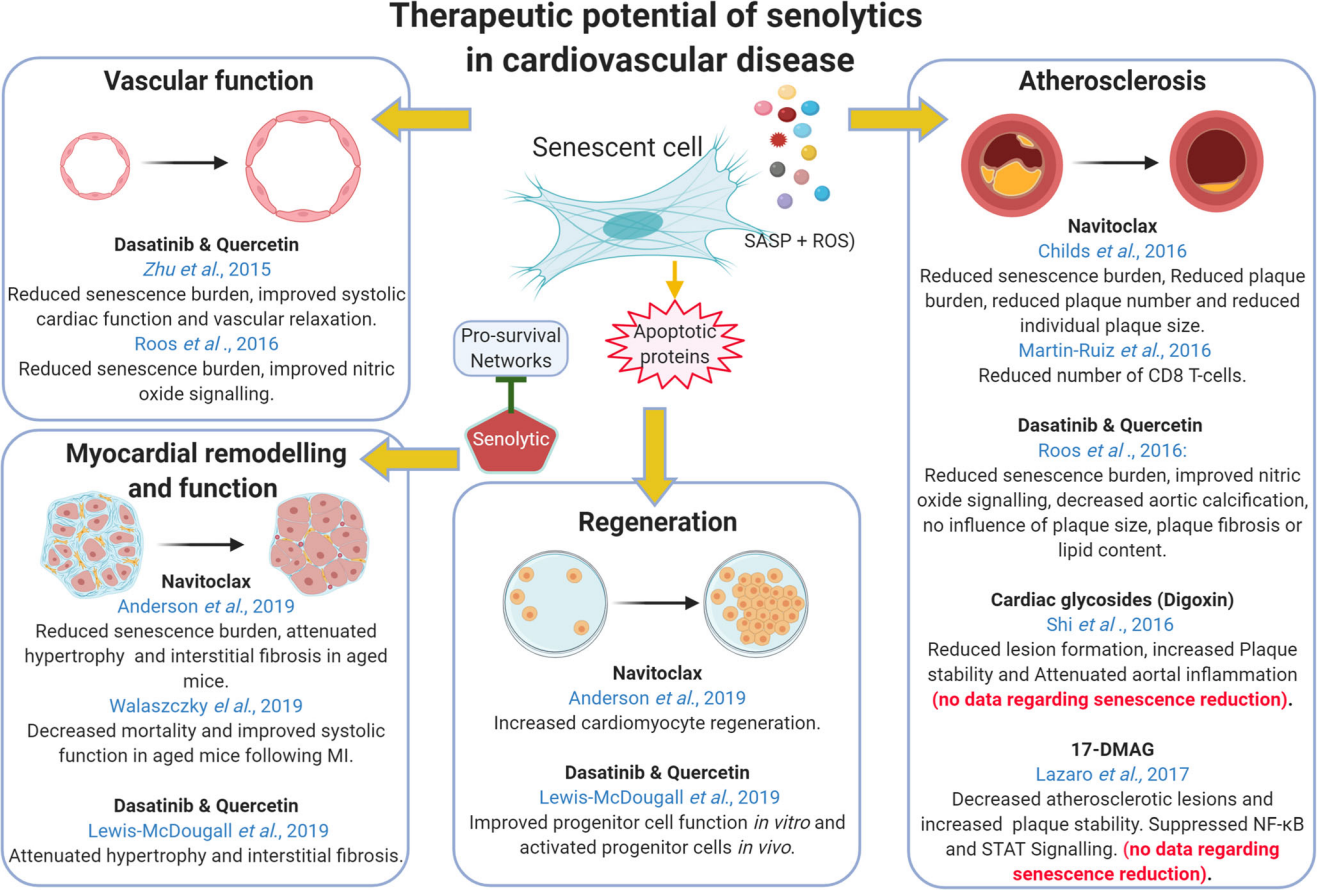
Therapeutic potential of senolytics in cardiovascular disease

While the above literature indicates that there is potential for senolytics to attenuate or prevent several CVD (Fig. 2) evidence suggests that senolytics can have adverse side effects. In particular, thrombocytopenia is a primary dose-limiting toxicity of navitoclax which is caused inhibition of Bcl-X_L_ within the platelet population, which is required for platelet survival [75]. D and Q also have several reported side effects, including hematologic dysfunction, fluid retention and QT prolongation [76].

Therefore, efforts are continuing to identify novel senolytics with safer profiles. In independent drug screens, both Triana-Martínez et al. [77] and Guerrero et al. [78] identified that cardiac glycosides (CG) are broad-spectrum senolytics. While the mechanisms whereby CG induces senescent cells to apoptosis are not yet fully understood, it has been suggested that CG bind to the alpha subunit of the Na^**+**^/K^**+**^ ATPase pump inhibiting the cellular intake of K^**+**^ and concurrently the release of Na^**+**^ enhancing plasma membrane depolarisation [77]. As senescent cells exhibit a partially depolarised plasma membrane, when compared with proliferating cells, they are more susceptible to the effects of CG [79]. However, CG treatment also resulted in elevated expression of pro-apoptotic Bcl-2 family members in oncogene-induced senescent fibroblasts, which could also contribute to their senolytic activities [78]. CG initiated senolysis in several models of oncogene and stress-induced senescence and eliminated senescent cells from the lungs of a xenograft model and an irradiation model of lung fibrosis. Furthermore, CG reduced senescence in the liver, heart and kidney of aged mice [77, 78]. Interestingly, the CG digoxin and digitoxin are currently used for treating HF and atrial fibrillation in elderly patients [80]. CG also reduced atherosclerosis in *Apo-E*-deficient mice [81], although these studies did not investigate if senolytic activity contributed to this anti-atherosclerotic effect. As mice treated with digitoxin showed no evidence of thrombocytopenia and as digitoxin behaves as a senolytic at concentrations close to those observed in the plasma of cardiac patients treated with this drug [82], Guerrero suggested the potential use of CG as senolytics in the clinic. It should however be highlighted that while current clinical guidelines endorse the use of digoxin in patients with atrial fibrillation, debate remains regarding its safety, and multiple studies have linked digoxin treatment with an increased relative risk of mortality [83–85].

Similarly, prior to the discovery of the senolytic properties of the HSP90 inhibitor 17-DMAG (alvespimycin) [86], it had been demonstrated that 17-DMAG reduced atherosclerosis in the *Apo-E* knock-out model, although it was originally assigned to be a result of NF-κB and STAT signalling pathway suppression [87]. It remains to be determined whether the senolytic activity of 17-DMAG also underlies its atherosclerosis limiting effects.

Senolytics with potentially limited side effects have been identified that show clinical promise including the FOXO4-D-retro-inverso (FOXO4-DRI) peptide which blocks the interaction of FOXO4 and p53 inducing senescent cells to apoptosis [88]. The senescence-specific killing compound 1 (SSK1) takes advantage of the increased activity of lysosomal β-galactosidase activity observed in senescent cells [89]. Derivatives or modified forms of navitoclax have also been generated to reduce toxicity. Ventoclax (ABT-199), a derivative of navitoclax, has a higher affinity to Bcl-2 and reduced affinity to Bcl-X_L_ Bcl-W and MCL-1, and thereby demonstrated decreased platelet toxicity [90]. In addition, He et al. have reduced the on-target toxicity of navitoclax by converting it into a platelet-sparing Bcl-xl proteolysis-targeting chimera (PROTAC), which contains a ligand that recruits a specific E3 ubiquitin ligase to induce protein degradation in a sub-stoichiometric manner resulting in less drug exposure and reduced toxicity [91]. These drugs hold great potential but have yet to be tested in the context of CVD.

Senolytic therapies have the potential to treat a wide range of CVD and age-associated cardiovascular pathologies. CVD, where senescence is suggested to play a pathogenic role, include arterial dysfunction and arterial stiffening associated with hypertension [92] and aortic aneurysms [93, 94]. Senescence has even been suggested to promote blood clotting via the SASP and contribute to increased thrombotic events resulting in stroke in the older population [95]. These indications may be potential targets for senolytics therapies and merit further investigation.

## Clinical Potential of Senolytics

Following the preclinical evidence showing the efficacy of senolytics for the prevention or treatment of several CVD, studies have now begun to investigate the use of senolytics in the clinical setting. In an initial pilot study to evaluate the feasibility of serotherapies, a treatment regime of 3-day D&Q treatment per week over 3 weeks had both high retention and completion rates and was found to be safe in a cohort of 14 idiopathic pulmonary fibrosis patients [96]. Moreover, treatment significantly improved physical function as evaluated by an increase in 6-min walk distance and 4-m gait speed and a reduction in chair-stands time; however, no change in pulmonary function was observed [96]. While the study was not powered to detect significant changes in SASP expression, correlations were observed between improved physical function and decreased circulating SASP proteins. In a preliminary report from a phase 1 clinical trial of D&Q in individuals with diabetic kidney disease, it was observed that senolytic therapy decreased senescence burden in both adipose and epidermis which is associated with a decrease in circulating cytokines and MMP including IL1α, IL2, IL6, IL9 and MMP2, MMP9 and MMP12 [97].

Currently available senolytics do not target an individual molecule or cell type and as such senolytic therapy will inevitably influence total systemic senescence burden. While this may be advantageous because ageing-associated senescence is targeted as a whole, it also prioritises the need for further understanding of the mechanisms by which senolytics attenuate CVD. For example, does clearance of senescent cardiac cells or non-cardiac populations, such as those of the immune system, lead to the observed improvement in cardiovascular health in the pre-clinical studies. Furthermore, the longer-term effects of a systemic elimination of senescent cells in humans are not yet known. Likewise, although in the short-term clearance of senescent cardiomyocytes improved cardiac function [17, 29], further studies are required to ensure that clearance of this post-mitotic population has no long-term detrimental effects on tissue integrity. While these questions remain to be answered, senolytics have the potential to transform cardiovascular medicine.

## References

[1] Passos JF, Simillion C, Hallinan J, Wipat A, von Zglinicki T (2009). Cellular senescence: unravelling complexity. Age (Dordr).

[2] Campisi J (2013). Aging, cellular senescence, and cancer. Annu Rev Physiol.

[3] Childs BG, Durik M, Baker DJ, van Deursen JM (2015). Cellular senescence in aging and age-related disease: from mechanisms to therapy. Nat Med.

[4] Watanabe S, Kawamoto S, Ohtani N, Hara E (2017). Impact of senescence-associated secretory phenotype and its potential as a therapeutic target for senescence-associated diseases. Cancer Sci.

[5] Rodier F, Campisi J (2011). Four faces of cellular senescence. J Cell Biol.

[6] Acosta JC, Banito A, Wuestefeld T, Georgilis A, Janich P, Morton JP, Athineos D, Kang TW, Lasitschka F, Andrulis M, Pascual G, Morris KJ, Khan S, Jin H, Dharmalingam G, Snijders AP, Carroll T, Capper D, Pritchard C, Inman GJ, Longerich T, Sansom OJ, Benitah SA, Zender L, Gil J (2013). A complex secretory program orchestrated by the inflammasome controls paracrine senescence. Nat Cell Biol.

[7] Kuilman T, Michaloglou C, Vredeveld LCW, Douma S, van Doorn R, Desmet CJ, Aarden LA, Mooi WJ, Peeper DS (2008). Oncogene-induced senescence relayed by an interleukin-dependent inflammatory network. Cell.

[8] Coppe JP (2010). The senescence-associated secretory phenotype: the dark side of tumor suppression. Annu Rev Pathol.

[9] Coppé JP, Patil CK, Rodier F, Sun Y, Muñoz DP, Goldstein J, Nelson PS, Desprez PY, Campisi J (2008). Senescence-associated secretory phenotypes reveal cell-nonautonomous functions of oncogenic RAS and the p53 tumor suppressor. PLoS Biol.

[10] Childs BG, Gluscevic M, Baker DJ, Laberge RM, Marquess D, Dananberg J, van Deursen JM (2017). Senescent cells: an emerging target for diseases of ageing. Nat Rev Drug Discov.

[11] Olivieri F, Recchioni R, Marcheselli F, Abbatecola AM, Santini G, Borghetti G, Antonicelli R, Procopio AD (2013). Cellular senescence in cardiovascular diseases: potential age-related mechanisms and implications for treatment. Curr Pharm Des.

[12] Hayflick L, Moorhead PS (1961). The serial cultivation of human diploid cell strains. Exp Cell Res.

[13] Bodnar AG (1998). Extension of life-span by introduction of telomerase into normal human cells. Science.

[14] Harley CB, Futcher AB, Greider CW (1990). Telomeres shorten during ageing of human fibroblasts. Nature.

[15] van Deursen JM (2014). The role of senescent cells in ageing. Nature.

[16] Anderson R, Richardson GD, Passos JF (2018). Mechanisms driving the ageing heart. Exp Gerontol.

[17] Anderson R, Lagnado A, Maggiorani D, Walaszczyk A, Dookun E, Chapman J, et al. Length-independent telomere damage drives post-mitotic cardiomyocyte senescence. EMBO J. 2019;38. 10.15252/embj.2018100492 PMC6396144 30737259

[18] Fumagalli M, Rossiello F, Clerici M, Barozzi S, Cittaro D, Kaplunov JM, Bucci G, Dobreva M, Matti V, Beausejour CM, Herbig U, Longhese MP, d’Adda di Fagagna F (2012). Telomeric DNA damage is irreparable and causes persistent DNA-damage-response activation. Nat Cell Biol.

[19] Hewitt G, Jurk D, Marques FDM, Correia-Melo C, Hardy T, Gackowska A, Anderson R, Taschuk M, Mann J, Passos JF (2012). Telomeres are favoured targets of a persistent DNA damage response in ageing and stress-induced senescence. Nat Commun.

[20] Williams GC (1957). Pleiotropy, natural selection, and the evolution of senescence. Evolution.

[21] Demaria M, Ohtani N, Youssef SA, Rodier F, Toussaint W, Mitchell JR, Laberge RM, Vijg J, van Steeg H, Dollé MET, Hoeijmakers JHJ, de Bruin A, Hara E, Campisi J (2014). An essential role for senescent cells in optimal wound healing through secretion of PDGF-AA. Dev Cell.

[22] Muñoz-Espín D, Cañamero M, Maraver A, Gómez-López G, Contreras J, Murillo-Cuesta S, Rodríguez-Baeza A, Varela-Nieto I, Ruberte J, Collado M, Serrano M (2013). Programmed cell senescence during mammalian embryonic development. Cell.

[23] Storer M, Mas A, Robert-Moreno A, Pecoraro M, Ortells MC, di Giacomo V, Yosef R, Pilpel N, Krizhanovsky V, Sharpe J, Keyes WM (2013). Senescence is a developmental mechanism that contributes to embryonic growth and patterning. Cell.

[24] Gorgoulis V, Adams PD, Alimonti A, Bennett DC, Bischof O, Bishop C, Campisi J, Collado M, Evangelou K, Ferbeyre G, Gil J, Hara E, Krizhanovsky V, Jurk D, Maier AB, Narita M, Niedernhofer L, Passos JF, Robbins PD, Schmitt CA, Sedivy J, Vougas K, von Zglinicki T, Zhou D, Serrano M, Demaria M (2019). Cellular senescence: defining a path forward. Cell.

[25] Baker DJ, Childs BG, Durik M, Wijers ME, Sieben CJ, Zhong J, A. Saltness R, Jeganathan KB, Verzosa GC, Pezeshki A, Khazaie K, Miller JD, van Deursen JM (2016). Naturally occurring p16Ink4a-positive cells shorten healthy lifespan. Nature.

[26] Baker DJ, Wijshake T, Tchkonia T, LeBrasseur NK, Childs BG, van de Sluis B, Kirkland JL, van Deursen JM (2011). Clearance of p16Ink4a-positive senescent cells delays ageing-associated disorders. Nature.

[27] Childs BG, Baker DJ, Wijshake T, Conover CA, Campisi J, van Deursen JM (2016). Senescent intimal foam cells are deleterious at all stages of atherosclerosis. Science.

[28] Demaria M, O'Leary MN, Chang J, Shao L, Liu S, Alimirah F, Koenig K, le C, Mitin N, Deal AM, Alston S, Academia EC, Kilmarx S, Valdovinos A, Wang B, de Bruin A, Kennedy BK, Melov S, Zhou D, Sharpless NE, Muss H, Campisi J (2017). Cellular senescence promotes adverse effects of chemotherapy and cancer relapse. Cancer Discov.

[29] Walaszczyk A, Dookun E, Redgrave R, Tual-Chalot S, Victorelli S, Spyridopoulos I, Owens A, Arthur HM, Passos JF, Richardson GD (2019). *Pharmacological clearance of senescent cells improves survival and recovery in aged mice following acute myocardial infarction*. Aging Cell.

[30] Lewis-McDougall FC, Ruchaya PJ, Domenjo-Vila E, Shin Teoh T, Prata L, Cottle BJ, Clark JE, Punjabi PP, Awad W, Torella D, Tchkonia T, Kirkland JL, Ellison-Hughes GM (2019). Aged-senescent cells contribute to impaired heart regeneration. Aging Cell.

[31] Zhu Y, Tchkonia T, Pirtskhalava T, Gower AC, Ding H, Giorgadze N, Palmer AK, Ikeno Y, Hubbard GB, Lenburg M, O'Hara SP, LaRusso NF, Miller JD, Roos CM, Verzosa GC, LeBrasseur NK, Wren JD, Farr JN, Khosla S, Stout MB, McGowan SJ, Fuhrmann-Stroissnigg H, Gurkar AU, Zhao J, Colangelo D, Dorronsoro A, Ling YY, Barghouthy AS, Navarro DC, Sano T, Robbins PD, Niedernhofer LJ, Kirkland JL (2015). The Achilles' heel of senescent cells: from transcriptome to senolytic drugs. Aging Cell.

[32] Soto-Gamez A, Quax WJ, Demaria M (2019). Regulation of survival networks in senescent cells: from mechanisms to interventions. J Mol Biol.

[33] Tchkonia T, Morbeck DE, von Zglinicki T, van Deursen J, Lustgarten J, Scrable H, Khosla S, Jensen MD, Kirkland JL (2010). Fat tissue, aging, and cellular senescence. Aging Cell.

[34] Kirkland JL, Tchkonia T (2017). Cellular senescence: a translational perspective. EBioMedicine.

[35] Short S, Fielder E, Miwa S, von Zglinicki T (2019). Senolytics and senostatics as adjuvant tumour therapy. Ebiomedicine.

[36] Tse C, Shoemaker AR, Adickes J, Anderson MG, Chen J, Jin S, Johnson EF, Marsh KC, Mitten MJ, Nimmer P, Roberts L, Tahir SK, Xiao Y, Yang X, Zhang H, Fesik S, Rosenberg SH, Elmore SW (2008). ABT-263: a potent and orally bioavailable Bcl-2 family inhibitor. Cancer Res.

[37] Olave NC, Grenett MH, Cadeiras M, Grenett HE, Higgins PJ (2010). Upstream stimulatory factor-2 mediates quercetin-induced suppression of PAI-1 gene expression in human endothelial cells. J Cell Biochem.

[38] Bruning A (2013). Inhibition of mTOR signaling by quercetin in cancer treatment and prevention. Anti Cancer Agents Med Chem.

[39] Roos CM, Zhang B, Palmer AK, Ogrodnik MB, Pirtskhalava T, Thalji NM, Hagler M, Jurk D, Smith LA, Casaclang-Verzosa G, Zhu Y, Schafer MJ, Tchkonia T, Kirkland JL, Miller JD (2016). Chronic senolytic treatment alleviates established vasomotor dysfunction in aged or atherosclerotic mice. Aging Cell.

[40] Farr JN, Xu M, Weivoda MM, Monroe DG, Fraser DG, Onken JL, Negley BA, Sfeir JG, Ogrodnik MB, Hachfeld CM, LeBrasseur NK, Drake MT, Pignolo RJ, Pirtskhalava T, Tchkonia T, Oursler MJ, Kirkland JL, Khosla S (2017). Corrigendum: targeting cellular senescence prevents age-related bone loss in mice. Nat Med.

[41] Ogrodnik M, et al. Cellular senescence drives age-dependent hepatic steatosis. Nat Commun. 2017;15691(8). 10.1038/ncomms15691 PMC5474745 28608850

[42] Schafer MJ, White TA, Iijima K, Haak AJ, Ligresti G, Atkinson EJ, Oberg AL, Birch J, Salmonowicz H, Zhu Y, Mazula DL, Brooks RW, Fuhrmann-Stroissnigg H, Pirtskhalava T, Prakash YS, Tchkonia T, Robbins PD, Aubry MC, Passos JF, Kirkland JL, Tschumperlin DJ, Kita H, LeBrasseur NK (2017). Cellular senescence mediates fibrotic pulmonary disease. Nat Commun.

[43] Xu M, Pirtskhalava T, Farr JN, Weigand BM, Palmer AK, Weivoda MM, Inman CL, Ogrodnik MB, Hachfeld CM, Fraser DG, Onken JL, Johnson KO, Verzosa GC, Langhi LGP, Weigl M, Giorgadze N, LeBrasseur NK, Miller JD, Jurk D, Singh RJ, Allison DB, Ejima K, Hubbard GB, Ikeno Y, Cubro H, Garovic VD, Hou X, Weroha SJ, Robbins PD, Niedernhofer LJ, Khosla S, Tchkonia T, Kirkland JL (2018). Senolytics improve physical function and increase lifespan in old age. Nat Med.

[44] Ogrodnik M, Zhu Y, Langhi LGP, Tchkonia T, Krüger P, Fielder E, Victorelli S, Ruswhandi RA, Giorgadze N, Pirtskhalava T, Podgorni O, Enikolopov G, Johnson KO, Xu M, Inman C, Palmer AK, Schafer M, Weigl M, Ikeno Y, Burns TC, Passos JF, von Zglinicki T, Kirkland JL, Jurk D (2019). Obesity-induced cellular senescence drives anxiety and impairs neurogenesis (vol 29, pg 1061, 2019). Cell Metab.

[45] Zhu Y, Tchkonia T, Fuhrmann-Stroissnigg H, Dai HM, Ling YY, Stout MB, Pirtskhalava T, Giorgadze N, Johnson KO, Giles CB, Wren JD, Niedernhofer LJ, Robbins PD, Kirkland JL (2016). Identification of a novel senolytic agent, navitoclax, targeting the Bcl-2 family of anti-apoptotic factors. Aging Cell.

[46] Chang J, Wang Y, Shao L, Laberge RM, Demaria M, Campisi J, Janakiraman K, Sharpless NE, Ding S, Feng W, Luo Y, Wang X, Aykin-Burns N, Krager K, Ponnappan U, Hauer-Jensen M, Meng A, Zhou D (2016). Clearance of senescent cells by ABT263 rejuvenates aged hematopoietic stem cells in mice. Nat Med.

[47] Pan J, Li D, Xu Y, Zhang J, Wang Y, Chen M, Lin S, Huang L, Chung EJ, Citrin DE, Wang Y, Hauer-Jensen M, Zhou D, Meng A (2017). Inhibition of Bcl-2/xl with ABT-263 selectively kills senescent type II pneumocytes and reverses persistent pulmonary fibrosis induced by ionizing radiation in mice. Int J Radiat Oncol Biol Phys.

[48] North BJ, Sinclair DA (2012). The intersection between aging and cardiovascular disease. Circ Res.

[49] Strait JB, Lakatta EG (2012). Aging-associated cardiovascular changes and their relationship to heart failure. Heart Fail Clin.

[50] Chimenti C, Kajstura J, Torella D, Urbanek K, Heleniak H, Colussi C, di Meglio F, Nadal-Ginard B, Frustaci A, Leri A, Maseri A, Anversa P (2003). Senescence and death of primitive cells and myocytes lead to premature cardiac aging and heart failure. Circ Res.

[51] Borlaug BA (2014). The pathophysiology of heart failure with preserved ejection fraction. Nat Rev Cardiol.

[52] Kadow ZA, Martin JF (2018). Distinguishing cardiomyocyte division from binucleation. Circ Res.

[53] Richardson GD, Laval S, Owens WA (2015). Cardiomyocyte regeneration in the mdx mouse model of nonischemic cardiomyopathy. Stem Cells Dev.

[54] Richardson GD. Simultaneous assessment of cardiomyocyte DNA synthesis and ploidy: a method to assist quantification of cardiomyocyte regeneration and turnover. J Vis Exp. 2016;111. 10.3791/53979 PMC4927713 27285379

[55] Bergmann O, Zdunek S, Felker A, Salehpour M, Alkass K, Bernard S, Sjostrom SL, Szewczykowska M, Jackowska T, dos Remedios C, Malm T, Andrä M, Jashari R, Nyengaard JR, Possnert G, Jovinge S, Druid H, Frisén J (2015). Dynamics of cell generation and turnover in the human heart. Cell.

[56] Abdelwahid E, Siminiak T, Cesar Guarita-Souza L, Athayde Teixeira de Carvalho K, Gallo P, Shim W, Condorelli G (2011). Stem cell therapy in heart diseases: a review of selected new perspectives, practical considerations and clinical applications. Curr Cardiol Rev.

[57] Malliaras K, Zhang Y, Seinfeld J, Galang G, Tseliou E, Cheng K, Sun B, Aminzadeh M, Marbán E (2013). Cardiomyocyte proliferation and progenitor cell recruitment underlie therapeutic regeneration after myocardial infarction in the adult mouse heart. EMBO Mol Med.

[58] Ellison GM, Vicinanza C, Smith AJ, Aquila I, Leone A, Waring CD, Henning BJ, Stirparo GG, Papait R, Scarfò M, Agosti V, Viglietto G, Condorelli G, Indolfi C, Ottolenghi S, Torella D, Nadal-Ginard B (2013). Adult c-kit(pos) cardiac stem cells are necessary and sufficient for functional cardiac regeneration and repair. Cell.

[59] Yazdanyar A, Newman AB (2009). The burden of cardiovascular disease in the elderly: morbidity, mortality, and costs. Clin Geriatr Med.

[60] McHugh D, Gil J (2018). Senescence and aging: causes, consequences, and therapeutic avenues. J Cell Biol.

[61] Oldershaw R, Owens WA, Sutherland R, Linney M, Liddle R, Magana L, Lash GE, Gill JH, Richardson G, Meeson A (2019). Human cardiac mesenchymal stem cell like cells, a novel cell population with therapeutic potential. Stem Cells Dev.

[62] Maggioni AA, Maseri A, Fresco C, Franzosi MG, Mauri F, Santoro E, Tognoni G (1993). Age-related increase in mortality among patients with first myocardial infarctions treated with thrombolysis. N Engl J Med.

[63] García Rubira JC, Valverde B, Romero D, García Martínez JT, López V, Rojas J, Ribas J, Pavón M, González M, Cruz Fernández JM (1995). Age is the independent prognostic factor in acute myocardial infarct. The clinical course of infarct in the elderly patient. An Med Interna.

[64] Boyle AJ, Hwang J, Ye J, Shih H, Jun K, Zhang Y, Fang Q, Sievers R, Yeghiazarians Y, Lee RJ (2013). The effects of aging on apoptosis following myocardial infarction. Cardiovasc Ther.

[65] Meyer K, Hodwin B, Ramanujam D, Engelhardt S, Sarikas A (2016). Essential role for premature senescence of Myofibroblasts in myocardial fibrosis. J Am Coll Cardiol.

[66] Zhu FL, et al. Senescent cardiac fibroblast is critical for cardiac fibrosis after myocardial infarction. PLoS One. 2013;8(9). 10.1371/journal.pone.0074535 PMC3770549 24040275

[67] Song P, Zhao Q, Zou M-H (2020). Targeting senescent cells to attenuate cardiovascular disease progression. Ageing Res Rev.

[68] Stojanović SD, Fiedler J, Bauersachs J, Thum T, Sedding DG (2020). Senescence-induced inflammation: an important player and key therapeutic target in atherosclerosis. Eur Heart J.

[69] Martin-Ruiz C (2020). CMV-independent increase in CD27−CD28+ CD8+ EMRA T cells is inversely related to mortality in octogenarians. npj Aging and Mechanisms of Disease.

[70] Callender LA, Carroll EC, Beal RWJ, Chambers ES, Nourshargh S, Akbar AN, Henson SM (2018). Human CD8+ EMRA T cells display a senescence-associated secretory phenotype regulated by p38 MAPK. Aging Cell.

[71] Ait-Oufella H, Salomon BL, Potteaux S, Robertson AKL, Gourdy P, Zoll J, Merval R, Esposito B, Cohen JL, Fisson S, Flavell RA, Hansson GK, Klatzmann D, Tedgui A, Mallat Z (2006). Natural regulatory T cells control the development of atherosclerosis in mice. Nat Med.

[72] Weng NP, Levine BL, June CH, Hodes RJ (1996). Regulated expression of telomerase activity in human T lymphocyte development and activation. J Exp Med.

[73] Richardson GD, Sage A, Bennaceur K, al Zhrany N, Coelho-Lima J, Dookun E, Draganova L, Saretzki G, Breault DT, Mallat Z, Spyridopoulos I (2018). Telomerase mediates lymphocyte proliferation but not the atherosclerosis-suppressive potential of regulatory T-cells. Arterioscler Thromb Vasc Biol.

[74] de Jesus BB, Schneeberger K, Vera E, Tejera A, Harley CB, Blasco MA (2011). The telomerase activator TA-65 elongates short telomeres and increases health span of adult/old mice without increasing cancer incidence. Aging Cell.

[75] Schoenwaelder SM, Jarman KE, Gardiner EE, Hua M, Qiao J, White MJ, Josefsson EC, Alwis I, Ono A, Willcox A, Andrews RK, Mason KD, Salem HH, Huang DCS, Kile BT, Roberts AW, Jackson SP (2011). Bcl-xL-inhibitory BH3 mimetics can induce a transient thrombocytopathy that undermines the hemostatic function of platelets. Blood.

[76] Breccia M, Molica M, Alimena G (2014). How tyrosine kinase inhibitors impair metabolism and endocrine system function: a systematic updated review. Leuk Res.

[77] Triana-Martínez F, Picallos-Rabina P, da Silva-Álvarez S, Pietrocola F, Llanos S, Rodilla V, Soprano E, Pedrosa P, Ferreirós A, Barradas M, Hernández-González F, Lalinde M, Prats N, Bernadó C, González P, Gómez M, Ikonomopoulou MP, Fernández-Marcos PJ, García-Caballero T, del Pino P, Arribas J, Vidal A, González-Barcia M, Serrano M, Loza MI, Domínguez E, Collado M (2019). Identification and characterization of cardiac glycosides as senolytic compounds. Nat Commun.

[78] Guerrero A, Herranz N, Sun B, Wagner V, Gallage S, Guiho R, Wolter K, Pombo J, Irvine EE, Innes AJ, Birch J, Glegola J, Manshaei S, Heide D, Dharmalingam G, Harbig J, Olona A, Behmoaras J, Dauch D, Uren AG, Zender L, Vernia S, Martínez-Barbera JP, Heikenwalder M, Withers DJ, Gil J (2019). Cardiac glycosides are broad-spectrum senolytics. Nat Metab.

[79] Warnier M, Flaman JM, Chouabe C, Wiel C, Gras B, Griveau A, Blanc E, Foy JP, Mathot P, Saintigny P, van Coppenolle F, Vindrieux D, Martin N, Bernard D (2018). The SCN9A channel and plasma membrane depolarization promote cellular senescence through Rb pathway. Aging Cell.

[80] Cheng JW, Rybak I (2010). Use of digoxin for heart failure and atrial fibrillation in elderly patients. Am J Geriatr Pharmacother.

[81] Shi H, Mao X, Zhong Y, Liu Y, Zhao X, Yu K, Zhu R, Wei Y, Zhu J, Sun H, Mao Y, Zeng Q (2016). Digoxin reduces atherosclerosis in apolipoprotein E-deficient mice. Br J Pharmacol.

[82] López-Lázaro M (2007). Digitoxin as an anticancer agent with selectivity for cancer cells: possible mechanisms involved. Expert Opin Ther Targets.

[83] Lopes RD, Rordorf R, de Ferrari GM, Leonardi S, Thomas L, Wojdyla DM, Ridefelt P, Lawrence JH, de Caterina R, Vinereanu D, Hanna M, Flaker G, al-Khatib SM, Hohnloser SH, Alexander JH, Granger CB, Wallentin L, ARISTOTLE Committees and Investigators (2018). Digoxin and mortality in patients with atrial fibrillation. J Am Coll Cardiol.

[84] Vamos M, Erath JW, Hohnloser SH (2015). Digoxin-associated mortality: a systematic review and meta-analysis of the literature. Eur Heart J.

[85] Vamos M, Erath JW, Benz AP, Lopes RD, Hohnloser SH (2019). Meta-analysis of effects of digoxin on survival in patients with atrial fibrillation or heart failure: an update. Am J Cardiol.

[86] Fuhrmann-Stroissnigg H, Ling YY, Zhao J, McGowan SJ, Zhu Y, Brooks RW, Grassi D, Gregg SQ, Stripay JL, Dorronsoro A, Corbo L, Tang P, Bukata C, Ring N, Giacca M, Li X, Tchkonia T, Kirkland JL, Niedernhofer LJ, Robbins PD (2017). Identification of HSP90 inhibitors as a novel class of senolytics. Nat Commun.

[87] Lazaro I, Oguiza A, Recio C, Mallavia B, Madrigal-Matute J, Blanco J, Egido J, Martin-Ventura JL, Gomez-Guerrero C (2015). Targeting HSP90 ameliorates nephropathy and atherosclerosis through suppression of NF-κB and STAT signaling pathways in diabetic mice. Diabetes.

[88] Baar MP (2017). Targeted apoptosis of senescent cells restores tissue homeostasis in response to chemotoxicity and aging. Cell.

[89] Cai Y, Zhou H, Zhu Y, Sun Q, Ji Y, Xue A, Wang Y, Chen W, Yu X, Wang L, Chen H, Li C, Luo T, Deng H (2020). Elimination of senescent cells by β-galactosidase-targeted prodrug attenuates inflammation and restores physical function in aged mice. Cell Res.

[90] Souers AJ, Leverson JD, Boghaert ER, Ackler SL, Catron ND, Chen J, Dayton BD, Ding H, Enschede SH, Fairbrother WJ, Huang DCS, Hymowitz SG, Jin S, Khaw SL, Kovar PJ, Lam LT, Lee J, Maecker HL, Marsh KC, Mason KD, Mitten MJ, Nimmer PM, Oleksijew A, Park CH, Park CM, Phillips DC, Roberts AW, Sampath D, Seymour JF, Smith ML, Sullivan GM, Tahir SK, Tse C, Wendt MD, Xiao Y, Xue JC, Zhang H, Humerickhouse RA, Rosenberg SH, Elmore SW (2013). ABT-199, a potent and selective BCL-2 inhibitor, achieves antitumor activity while sparing platelets. Nat Med.

[91] He Y, Zhang X, Chang J, Kim HN, Zhang P, Wang Y, Khan S, Liu X, Zhang X, Lv D, Song L, Li W, Thummuri D, Yuan Y, Wiegand JS, Ortiz YT, Budamagunta V, Elisseeff JH, Campisi J, Almeida M, Zheng G, Zhou D (2020). Using proteolysis-targeting chimera technology to reduce navitoclax platelet toxicity and improve its senolytic activity. Nat Commun.

[92] Durik M, Kavousi M, van der Pluijm I, Isaacs A, Cheng C, Verdonk K, Loot AE, Oeseburg H, Bhaggoe UM, Leijten F, van Veghel R, de Vries R, Rudez G, Brandt R, Ridwan YR, van Deel ED, de Boer M, Tempel D, Fleming I, Mitchell GF, Verwoert GC, Tarasov KV, Uitterlinden AG, Hofman A, Duckers HJ, van Duijn CM, Oostra BA, Witteman JCM, Duncker DJ, Danser AHJ, Hoeijmakers JH, Roks AJM (2012). Nucleotide excision DNA repair is associated with age-related vascular dysfunction. Circulation.

[93] Cafueri G, Parodi F, Pistorio A, Bertolotto M, Ventura F, Gambini C, Bianco P, Dallegri F, Pistoia V, Pezzolo A, Palombo D (2012). Endothelial and smooth muscle cells from abdominal aortic aneurysm have increased oxidative stress and telomere attrition. PLoS One.

[94] Balint B, Yin H, Nong Z, Arpino JM, O'Neil C, Rogers SR, Randhawa VK, Fox SA, Chevalier J, Lee JJ, Chu MWA, Pickering JG (2019). Seno-destructive smooth muscle cells in the ascending aorta of patients with bicuspid aortic valve disease. EBioMedicine.

[95] Wiley CD (2019). SILAC Analysis Reveals Increased Secretion of Hemostasis-Related Factors by Senescent Cells. Cell Rep.

[96] Justice JN, et al. Senolytics in idiopathic pulmonary fibrosis: results from a first-in-human, open-label, pilot study. EBioMedicine. 2019. 10.1016/j.ebiom.2018.12.052 PMC6412088 30616998

[97] Hickson LJ, Langhi Prata LGP, Bobart SA, Evans TK, Giorgadze N, Hashmi SK, Herrmann SM, Jensen MD, Jia Q, Jordan KL, Kellogg TA, Khosla S, Koerber DM, Lagnado AB, Lawson DK, LeBrasseur NK, Lerman LO, McDonald KM, McKenzie TJ, Passos JF, Pignolo RJ, Pirtskhalava T, Saadiq IM, Schaefer KK, Textor SC, Victorelli SG, Volkman TL, Xue A, Wentworth MA, Wissler Gerdes EO, Zhu Y, Tchkonia T, Kirkland JL (2019). Senolytics decrease senescent cells in humans: preliminary report from a clinical trial of Dasatinib plus Quercetin in individuals with diabetic kidney disease. EBioMedicine.

